# SiamHAS: Siamese Tracker with Hierarchical Attention Strategy for Aerial Tracking

**DOI:** 10.3390/mi14040893

**Published:** 2023-04-21

**Authors:** Faxue Liu, Jinghong Liu, Qiqi Chen, Xuan Wang, Chenglong Liu

**Affiliations:** 1 Changchun Institute of Optics, Fine Mechanics and Physics (CIOMP), Chinese Academy of Sciences, Changchun 130033, China; 2 University of Chinese Academy of Sciences, Beijing 100049, China

**Keywords:** Siamese tracker, deep learning, hierarchical attention strategy, multi-level feature enhancement

## Abstract

For the Siamese network-based trackers utilizing modern deep feature extraction networks without taking full advantage of the different levels of features, tracking drift is prone to occur in aerial scenarios, such as target occlusion, scale variation, and low-resolution target tracking. Additionally, the accuracy is low in challenging scenarios of visual tracking, which is due to the imperfect utilization of features. To improve the performance of the existing Siamese tracker in the above-mentioned challenging scenes, we propose a Siamese tracker based on Transformer multi-level feature enhancement with a hierarchical attention strategy. The saliency of the extracted features is enhanced by the process of Transformer Multi-level Enhancement; the application of the hierarchical attention strategy makes the tracker adaptively notice the target region information and improve the tracking performance in challenging aerial scenarios. Meanwhile, we conducted extensive experiments and qualitative or quantitative discussions on UVA123, UAV20L, and OTB100 datasets. Finally, the experimental results show that our SiamHAS performs favorably against several state-of-the-art trackers in these challenging scenarios.

## 1. Introduction

In the past decade, deep learning methods have been widely used and driven innovation in various research fields [1,2,3,4]. As an important task in the field of applications in deep learning, target tracking can be cross-applied with many disciplines, making it a hot topic of interest for researchers in many related fields, and is widely used in intelligent video surveillance [5], UAV applications [6], and assisted autonomous driving [7]. The task of traditional target tracking can be described as the following process: given only the initial location information and size of an arbitrary target in a video segment, the tracker needs to continuously estimate the subsequent motion state of the target without using other prior information. However, the actual video sequences often consist of scenarios such as scale variation, occlusion, and low resolution, which require high accuracy and robustness of the trackers, and it is a challenging task to ensure the performance of the tracker in complex scenes.

The existing target tracking methods could be divided into generative model-based tracking methods [8,9] and discriminative model-based tracking methods [10,11] according to the method of building target features. The former refers to the traditional methods which use the target’s feature and rely on manually designed features, such as the optical flow method [12], mean drift [13], etc., with generally poor tracking results. The latter refers to the discriminative model-based tracking methods that exploit both target motion foreground and background information, which have become mainstream over the last decade. The foreground indicates the pixels’ location where the target region in the feature maps is located, and the background refers to the pixel area occupied by the target motion background. Correctly distinguishing the foreground from the background is the key factor to locate the target object location and generate the predicted banding box. A step further, according to the implementation principle, it can be divided into correlation filter-based [14,15,16,17] and deep learning-based [18,19,20,21] tracking models. The key step in the correlation filter approach is to obtain the filter with optimal performance so that the search area and the target area could generate an accurate maximum response. For example, the KCF tracker [22], which is the cornerstone of the correlation filtering algorithms family, uses a similarity-matching strategy that transforms the image-matching task into a classification task of foreground and background and reduces the computational effort of the algorithm by using circular matrices and fast Fourier transforms. However, it cannot meet the requirements of tracking accuracy in fast background movement, small target object tracking, or other aerial scenarios. Among the features outputted by the deep learning-based feature extraction network, the shallow features contain location information, and in this regard, the shallower feature extraction network is more beneficial for determining the target position. The deeper features contain rich semantic information about the foreground and background in the images, which is useful for tracking in scenarios such as motion blur, low resolution, etc. The deep learning-based approach can automatically extract features at different depth levels through the feature extraction network and can effectively utilize the background information of the target motion, which makes it have strong feature extraction capability and robustness compared to traditional methods and correlation filter-based methods. In recent years, Siamese neural network-based trackers [23,24,25,26] have received extensive attention from researchers in the field.

Based on the above analysis, we propose a novel Siamese tracker based on the feature enhancement module of Transformer with the hierarchical attention strategy. The algorithm is mainly composed of the following four parts: feature extraction, feature fusion, similarity matching, and classification regression prediction head. The hierarchical attention strategy is the main characteristic of the feature fusion network. The self-attention mechanism can effectively improve the saliency of each branch, and the hierarchical attention strategy could achieve feature enhancement at different levels so that the features could include global contextual information and make it easier to utilize the features effectively. Experiments demonstrate that our SiamHAS can effectively enhance the tracking performance of Siamese neural networks in challenging scenarios.

The main contributions of our proposed approach can be summarized as follows:

1. We propose a target tracker with the strategy of hierarchical attention. After comparing with several state-of-the-art Siamese trackers, our SiamHAS reaps more stable robustness and precision in challenging scenes such as target occlusion, especially in various challenges in aerial scenarios, and achieves the desired goal.
2. In the feature fusion sub-network, a feature enhancement method integrating multiple attention strategies is introduced in this paper. With the channel context-aware mechanism, global context information can be included among different channels, which achieves feature enhancement in the channel dimension. The introduction of multiple modified Transformer encoders enhances the saliency of deeper features in each branch and effectively improves performance.
3. Extensive comparative experiments on our proposed SiamHAS are conducted on many benchmarks with complex and challenging scenarios. The experiments demonstrate that our algorithm obtains high robustness in challenging scenarios, and the hierarchical attention mechanism and Transformer module can strongly improve the performance of the Siamese network-based tracker.

## 2. Related Work

In this section, we briefly review the research related to our work in recent years, including a summary of the Siamese neural network trackers, attention mechanisms, and Transformers.

### 2.1. Siamese Trackers

The core idea of the Siamese trackers is to learn the information between the target template and the search region and then transform the tracking tasks into a similarity-matching problem between the two branches. The first Siamese tracker is SINT [27], and the subsequent series of Siamese trackers have a similar structure of using two branches of the same backbone network to generate feature maps. Then, after the mutual correlation operation, the best matching search region in the response maps is generated by the similarity matching network to complete the tracking, such as SiamFC [28], SiamDW [29], SiamDW-RPN [30], and SiamFC++ [31]. Inspired by Faster R-CNN [32] which proposed the RPN network to avoid the process of extracting feature maps at multiple scales, SiamRPN was proposed by Li et al. [33]. DaSiamRPN [34] achieves a degree of performance improvement but the depth of the extracted features is relatively shallow. SiamRPN++ [35] applies a deeper feature extraction network, but it uses a direct linear weighting fusion method without deeply exploring how to fully utilize the advantage of the deep network’s multiple layers feature. In recent years, more advanced methods such as HiFT [36], SiamBAN [37], SiamCAR [38], and Ocean [39] have been proposed one after another, all of which use deeper feature extraction networks. Although the above-mentioned trackers are applied to modern deep feature extraction networks, they do not take full advantage of the different levels of features, resulting in relatively poor performance in aerial scenarios, such as target occlusion, scale variation, and low-resolution target tracking. 

### 2.2. Attention Mechanism

#### 2.2.1. Introduction to Attention Mechanism

The attention mechanism [40,41,42] is one of the ways for humans to filter information efficiently. The main implementation of the attention mechanism can be described as follows: n input messages represented by a key-value pair can be written as [(k_1_,v_1_), (k_2_,v_2_)…, (k_n_,v_n_)]. The first step is the attention scoring function that uses the dot product model, which uses the inner product calculation to calculate the similarity of each covariate in the query object *Q* and *K*. In the second step, the attention scores are normalized to obtain the probability of each similarity, and the probability vector is called attention distribution. The normalization feature of the softmax function is used to highlight the weights of important elements and bring the network’s attention to the areas that need more attention. The overall implementation process of the attention mechanism can be summarized in the following equation. The attention score of each element in *Q* and *K* can be obtained as S. A is the final attention results, *Q* is the query variables, *K* is the key in the input key-value pair, and *V* is the value.

(1) A(Q,K,V)=softmax(S(Q,K))∗V

In the third step, the value is the weighted sum according to the coefficient of the attention distribution to aggregate information and obtain the final output result. The three-step process described above can be expressed as follows:

(2) $$\begin{array}{l} \mathrm{STEP}1:S=s_{1},s_{2},\ldots ,s_{n}=Q*K;(K=k_{1},k_{2},\ldots ,k_{n})\\ \mathrm{STEP}2:D=d_{1},d_{2},\ldots ,d_{n}=\mathrm{softmax}(s_{1},s_{2},\ldots ,s_{n})=\frac{exp(s_{i})}{\sum_{j=1}^{n}\left(s_{j}\right)}\\ \mathrm{STEP}3:A=\sum_{i=1}^{n}d_{i}v_{i} \end{array}$$

In this formula, the *K* represents the input keys in the key-value pair, in different conditions, it could be the original input values or input values after the linear transformation. S represents the attention score for the input key-value pair, *s_i_* represents the attention score for each element in the input, and n represents the number of elements in the input. D represents the attention distribution for the input key-value pair, and it could be calculated by the softmax function with the input of S where the result is a normalized distribution of S. *d_i_* represents the attention distribution for each element in the input. A represents the final attention results, it is the sum of all the products of *v_i_* which is the value element of all key-value pairs in the input and the corresponding attention distribution *d_i_*.

#### 2.2.2. Typical Attention Mechanisms in Computer Vision

**Figure 1:**
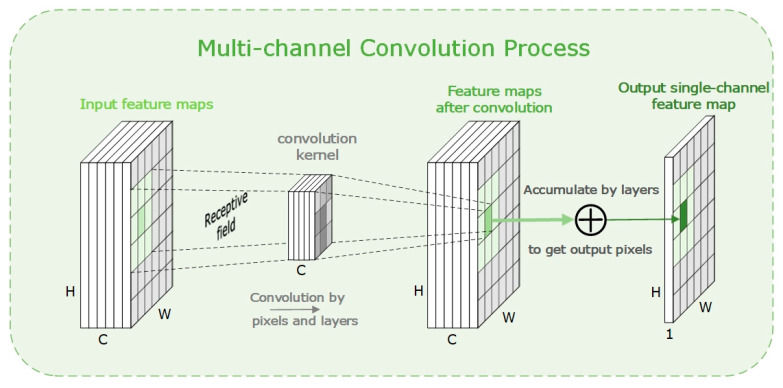
A multi-channel convolution process where a single convolutional kernel convolves with the corresponding feature maps, and the individual layer pixel positions are added to produce an output single-channel feature map.

Generally, self-attention [43] and non-self-attention are the typical classification method according to whether the ternary inputs are from the same source or not. In computer vision tasks, self-attention mechanisms are commonly used. According to the different dimensions of the extracted attention distribution, it can be generally classified into several forms of spatial [44], channel, and context [45] attention mechanisms. Taking the channel attention mechanism as an example, since the modern feature extraction network has deep layers, the change of channel dimension is essential, usually, the convolutional layers make significant contributions to the process, as shown in Figure 1.

The output feature map by each convolution kernel extracts a kind of feature of the target object, and each channel represents a matrix of each kind of feature. So, in the learning process, the parameters of the convolution kernel could adaptively vary and offer the net an appropriate structure. Similarly, the channel attention could finally obtain an attention distribution that can be recognized as a convolution kernel which could be optimized through the training process. 

### 2.3. Transformer and Multi-Head Attention

**Figure 2:**
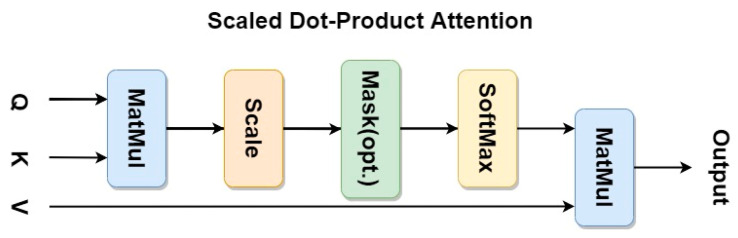
A typical self-attention mechanism implementation process where MatMul represents the dot product operation of the input matrix Q and K.

Distinct from the attention mechanism in a broad sense, the self-attention mechanism stipulates a new restriction that the three elements of the input QKV must be homologous, which is usually known as the Transformer structure [46]. A typical Transformer structure definition had been released in “Attention is all you need” by Ashish Vaswani [47] in 2017. Its main implementation process can be summarized as the process of Scaled Dot-Product Attention, which is shown in Figure 2.

The mathematical expression of the model could be expressed as

(3) $$\begin{array}{c} \mathrm{Attention}(Q,K,V)=\mathrm{softmax}(\frac{QK^{T}}{\sqrt{dk}})V\\ Query:Q=W^{Q}X\\ Key:K=W^{K}X\\ Value:V=W^{V}X \end{array}$$

**Figure 3:**
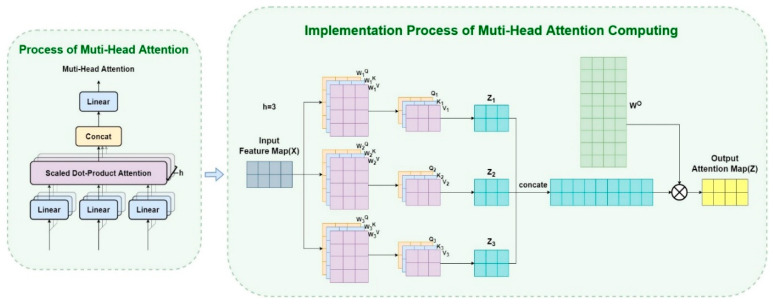
The implementation process of multi-head attention computing.

The matrix *X* represents the input data, and the values of *Q*, *K*, and *V* are derived from the same input *X* through matrix operations with three hyperparameters $W^{Q},W^{K},W^{V}$ that are first initialized and then learned through the process of optimization in training by applying an SGD optimizer with the strategy of random batch gradient descent. We use the dimension of the key, which is the number of its channels, as a parameter *d_k_* to scale the output of MatMul. This avoids the possibility of distortion in the softmax process which represents the process of obtaining a probability vector that stores attention distribution. The model of the multi-head attention mechanism describes a detailed introduction to the linear transformation matrix operations. The following Figure 3 and formulas illustrate the implementation process.

(4) $$\begin{array}{c} \mathrm{MultiHead}(Q,K,V)=\mathrm{Concat}({\mathrm{head}}_{1},\ldots ,{\mathrm{head}}_{h})W^{O}\\ \mathrm{headi}=\mathrm{Attention}(QW_{i}^{Q},KW_{i}^{K},VW_{i}^{V}) \end{array}$$

In the above formula, *Q*, *K*, and *V* represent query, key, and value, respectively. For each head, head_i_ is computed in the same process as the self-attention computation mentioned above, with each head initializing a different set of *W_i_* parameter matrices. Furthermore, the results of these heads are finally concatenated together and then it is multiplied with a trainable parameter matrix $W^{O}$ to generate the final multi-head attention output. The multi-head attention calculates the similarity between *Q* and *K*, so the attention map could be obtained. Then, through the process of multiplying *V* with the normalized attention map, finally, we finish the process of enhancement for the inputs. To improve the various deficiencies of the existing methods, reference [48] adopts a Transformer-based feature fusion structure, which establishes the connection of global context, and the critical step is the powerful ability of the multi-head self-attention in the core module of the Transformer encoder.

## 3. Proposed Approach

In our proposed approach, we specifically introduce the proposed Siamese tracker and detail the overall structural composition of the algorithm and the principle of implementation. We focus on the structural composition of the hierarchical attention strategy for feature enhancement of the feature fusion sub-network then detail its basic modules and the important influence of the algorithm structure design. In particular, we focus on the implementation of the fusion strategy with channel, context, and multi-headed attention mechanisms, and the improvement of each part of the module, the architectural design, and implementation principles are presented in detail. Finally, we analyze the architectural design advantages of our proposed algorithm from the top-level perspective of the network.

### 3.1. The Overall Structure of SiamHAS

**Figure 4:**
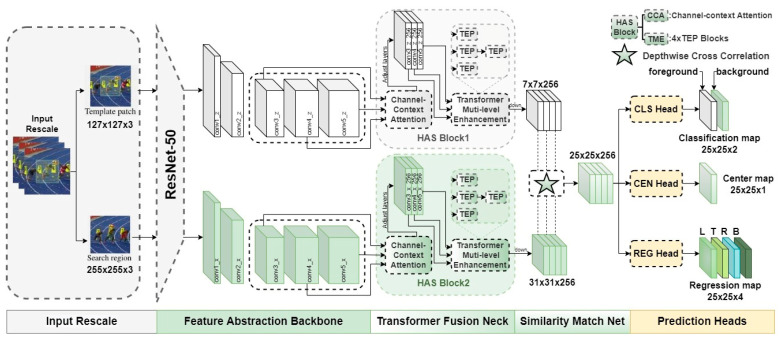
The overall framework of the proposed SiamHAS tracker.

The proposed algorithm consists of the following four main modules: feature extraction backbone, feature fusion and enhancement network, similarity matching network, and classification regression prediction heads. The overall framework of our proposed tracker in this paper is shown in Figure 4.

In the Transformer Fusion Neck part, we propose the hierarchical attention strategy block and abbreviate it as the HAS block. It consists of the proposed channel context-aware attention module, which is abbreviated as the CCA module, and the Transformer multi-level enhancement module, which is abbreviated as the TME module, to realize the global correlation enhancement before feature fusion. Specifically, the last three-layer feature blocks output by the backbone are respectively used as the QKV ternary inputs of the three-level CCA modules, and the optimized three-layer feature blocks with the same dimension are the output. The following is a detailed description of the proposed CCA module.

#### 3.1.1. The Proposed CCA Module: Channel Context-Aware Attention

**Figure 5:**
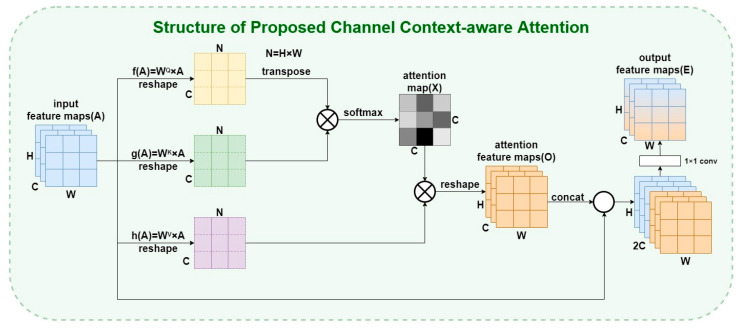
The structure design of the proposed CCA module.

For typical channel attention, if the dimension of the convolution output is C × H × W, the channel attention distribution learned through the network is stored in the C-dimensional vector, and the weight values of each layer are applied to all positions of each channel of the feature maps in turn. It could be the traditional way to realize the channel attention mechanism, but it still cannot make the most of the global context-aware information for modeling, and the efficacy is still comparatively low. Global context-aware information is a crucial factor for aerial tracking, as it could help to globally model using the correlation between consecutive frames to avoid tracking drift caused by temporary disappearance of the target in some frames or occlusion scenes. We propose a channel context-aware attention block applied to the fusion section, trying to apply the method of global contextual attention mechanism to different levels of features and realize the mining process of different depths of feature information from the dimension of channels. Specifically, we improve the optimization approach of attention feature maps in reference [45] from the perspective of theoretical analysis and experimental validation to balance the features at different levels. To adapt to the characteristics of the feature maps in aerial scenes and balance the effect of the information contained in different levels of feature maps, we concatenate the attention feature maps’ output with the initial input feature maps and adjust them with a 1 × 1 convolutional layer as the final output to achieve better feature utilization in aerial scenes and improve the performance of the tracker in challenging scenes. The channels containing the target features receive a larger attention scoring result through global contextual modeling to highlight the information of these channels and complete the pre-processing of the input data for the next process of Transformer multi-level enhancement. The specific implementation process of the proposed CCA module is displayed in Figure 5.

Specifically, the input feature maps are reshaped to obtain the new A in a different shape of C × N, which is then transposed to obtain A^T^ and perform matrix multiplication of the two obtained feature maps. Then the attention map X in the shape of C × C is obtained after passing through the softmax layer. Through the process of matrix multiplication of X^T^ and A in the shape of C×N, the attention scoring process can be applied to different channels based on different attention scores, and then reshape the results to the original shape to obtain attention feature maps O. The attention map involved in the calculation in this process could be expressed as follows:

(5) $$\begin{array}{l} X=\mathrm{softmax}({\left(A^{Q}\right)}^{T}\cdot A^{K})\\ x_{KQ}=\frac{exp(A^{Q}\cdot A^{K})}{\sum_{i=1}^{C}exp(A^{Q}\cdot A^{K})} \end{array}$$

where X represents the attention map, $A^{Q}$ and $A^{K}$ represent the features after the processing of the trainable parameter matrices $W^{Q}$ and $W^{K}$, respectively. $x_{KQ}$ represents each element of the attention map, corresponding to the attention value of each location point. The output O and *A* are then fused through a process of channel cascade, and finally, the final output E is obtained through 1*1 convolution. The operation could be represented as

(6) E=conv(concat(A,O))

(7) $$O=\mathrm{conv}(A^{V}\cdot X)$$

where E represents the final output feature maps, $A^{V}$ represents the features after the processing of the trainable parameter matrix $W^{V}$, O represents the attention feature maps after applying the attention map as a weight map to multiply with $A^{V}$, conv represents the convolution operation, and concat represents the process of the concatenation. By the process of concatenation of the original input and the attention feature maps, the final output could contain multi-level information. This is an essential strategy to cope with aerial scenes such as occlusion or low resolution, because it is extremely demanding to fuse deep semantic information and shallow spatial information to complete the tracking process. 

In summary, the proposed CCA module completes the optimization process of the source input data in the channel dimension by global modeling of the global context, using a self-attentive mechanism to obtain the channel correlations between any two different channels, and updating every channel graph using the strategy of the weighted sum of all channels.

After the tensor data is optimized by the CCA module, the feature information will be reduced by adjusting layers to avoid the large number of computational resources required in the subsequent TME module processing. Specifically, at the adjusting layers part, we take advantage of the multiple convolutional layers dimension adjustment, and the number of channels of the three-layer feature blocks after channel cascading is uniformly adjusted from the original [512, 1024, 2048] channels to [256, 256, 256] channels. This is to decrease the subsequent amount of parameters, and the dimensionality of the number of channels of the feature blocks will become 3 × 256. The following is a detailed description of the proposed TME module.

#### 3.1.2. The Proposed TME Module: Transformer Multi-Level Enhancement

It is generally believed that the ability to effectively utilize the depth advantage of modern deep feature extraction networks is the key step to enhancing the existing Siamese trackers, and one of the most important steps is to effectively utilize different levels of deep features. Among the features extracted by the backbone at different depth levels, the shallow layer output features retain more concrete spatial information, such as edge, color, texture, etc., which helps to locate the target frame. The high-level feature maps after multiple convolutional and pooling layers contain deep semantic information, which can help the classification network to determine whether each pixel location is foreground or background. How to effectively utilize the feature information at different levels is a crucial process to enhance the model’s performance. 

In aerial scenarios such as occlusion and motion blur, deep semantic feature information is detrimental to distinguishing the targets. In aerial tiny target detection and low-resolution scenarios, the pixel area of the target object has too few pixels to retain enough shallow spatial feature information, causing degradation in tracking performance. The existing methods often lack global interactions, and it is difficult to effectively exploit the association advantage of global contextual hierarchical features. To enhance the saliency of the hierarchical features at different levels to improve the performance of the tracker in the above aerial scenarios, we propose a TME module based on a hierarchical self-attentive strategy to cope with this problem. 

**Figure 6:**
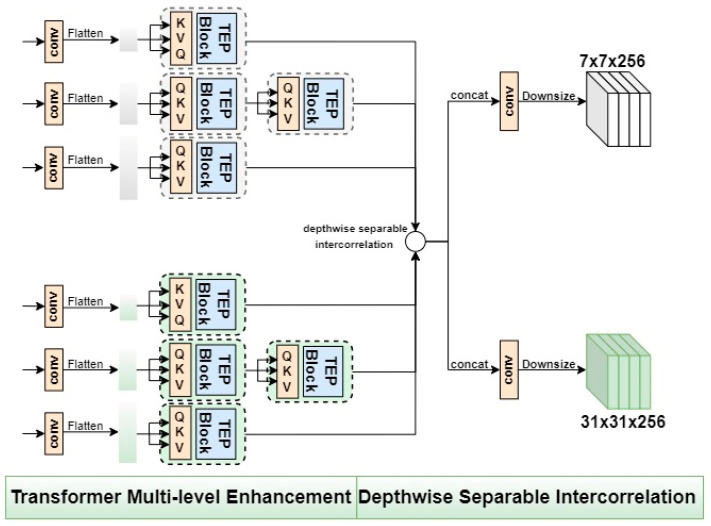
The schematic of the process of Transformer multi-level enhancement, depthwise separable intercorrelation, and downsize operation.

Specifically, the pooling-based encoder structure is modified as the TME module in the second core attention in our proposed hierarchical attention strategy. As shown in Figure 6, the average pooling strategy is used in the Transformer encoder with the pooling strategy block, which is abbreviated as the TEP block, as a pretreatment strategy for refining the input data of the *K* and *V* parameters. We modify the typical Transformer encoder structure proposed in reference [48] as the basic architecture of the proposed TEP module through theoretical analysis and several experimental adjustments. We use two tandem TEP structures in the conv4 branch and a single TEP structure in the remaining two branches to achieve a balanced utilization of the features at different levels. Finally, we integrate the TME module consisting of four TEP modules into the structure design of the tracker to achieve better tracking performance. To further optimize to obtain a lighter Transformer-based structure for object tracking tasks, we replace the process of position coding of the typical Transformer structure with the zero-padding strategy using the location information of the inputs to ensure the integrity of input information. 

The core process of the TEP block is processing the input ternary data, which is essentially the calculation process of the proposed pooling-based encoder, this could be mathematically expressed as

(8) $$\begin{array}{l} \mathrm{TEP}(Q,K,V)=\mathrm{Norm}(I+\mathrm{MLP}(I))\\ I=\mathrm{Norm}(Q+\mathrm{MutiHead}(Q,\mathrm{Norm}\&\mathrm{Pooling}(K,V))) \end{array}$$

where *QKV* represent the ternary inputs, which are query variables, key-value variables, and value variables of the key-value pairs, respectively. MLP represents a fully connected feed-forward network, Pooling represents average pooling, and Norm represents the process of LayerNorm for smoothing the inputs. The following is a detailed description of how the proposed TME module is applied in our SiamHAS structure. Specifically, as shown in Figure 6, after the dimension adjustment, the feature block of 3,4,5 of each layer is flattened into sequence information using the convolution operation, and they are used as the inputs of the subsequent Transformer multi-level enhancement module, respectively, as the *QKV* ternary input of the three TEP blocks. The results of the TEP block whose input is conv4 are sent to another TEP block to achieve a balance of deep-level and shallow-level features through the calculation of double-layer self-attention. The three-branch TEP-based feature optimization calculation process constitutes the transformer multi-level enhancement process of our TME module. Taking the process of obtaining the results of the conv4 feature block $F_{4}^{\prime }$ as an example, it could be mathematically expressed as

(9) $$\begin{array}{l} R=\mathrm{TEP}(F_{4},F_{4},F_{4})\\ F_{4}^{\prime }=\mathrm{TEP}(R,R,R) \end{array}$$

where the intermediate variable *R* represents the features processed by the first TEP module and is used as the ternary input for the second TEP module. 

#### 3.1.3. The Similarity Matching Net and Downsize Feature Fusion

The output of the HAS block is the hierarchical fusion features that consist of shallow spatial information and deep semantic information. Finally, the number of channels and pixels of the feature maps will be further reduced by the downsizing feature fusion process after the similarity matching process, which greatly reduces the computational quantity in the following procedures of the feature processing In the similarity matching sub-network, the depthwise separable intercorrelation operation is used to calculate the response maps after each level of intercorrelation through a hierarchical convolution of the corresponding layers of 3,4,5 of the target template and the search region. The above process can be described as

(10) R=φ(X)⋆φ(Z)

where ⋆ represents the depthwise separable intercorrelation operation, and $\varphi \left(X\right)$ and $\varphi \left(Z\right)$ represent the hierarchical convolutional inputs corresponding to layers of 3,4,5 of the search region and template branch, respectively, which is also could be regarded as the hierarchical three-layer outputs of the TME blocks in the search region and template branch. The number of channels of the response map output is 256 × 3, which is still too large if it is directly used as the input of the subsequent classification and regression network. Therefore, we utilize multiple convolutional layers to decrease the dimensionality and adjust the dimension of the response maps. Specifically, the three blocks at different levels after the depthwise separable intercorrelation are sent to the downsizing block, which consists of multiple convolutional layers, to obtain 7 × 7 × 256 tensor for the template patch branch and 31 × 31 × 256 for the search region branch as the inputs of the following prediction heads. This operation decreases the number of model parameters and improves the speed of model derivation, ensuring a balance between model effectiveness and computational efficiency to the greatest extent. The above process can be represented by the following Figure 6.

#### 3.1.4. The Prediction Heads

The prediction heads consist of three branches, including the CLS head, CEN head, and REG head shown in Figure 4, where the CLS head represents the classification network, the REG head represents the regression network, and the CEN head represents the centrality network. The role of the CLS head is to determine the attribute of every pixel position using the binary attributes of the target and background, to predict the attribute of the feature map location. The CEN head could obtain the prediction box’s centroid through multiple convolutional layers. The REG head could obtain the prediction box’s width and height to generate the tracking banding box through the continuous regression calculation of the network. Finally, the foreground and background are distinguished by the classification and regression network to obtain the target prediction frame. In the prediction head network, the response map generated after the intercorrelation operation can be regarded as a linear mapping of the input image in the space under the weight of the target template, and each position of the response map could be mapped to the corresponding position in the original image space of the search region, and the crucial process of mapping is the size of the receptive field of the linear transformation. Different from the algorithm based on region proposal network that uses fixed multi-scale frames for similarity matching to obtain multiple response maps, and then maps its maximum response area back to the corresponding search region position to achieve the prediction frame centroid and output the prediction frame by size regression, in this paper, we adopt the anchor-free mechanism to directly classify and regress the candidate frames at each position without setting a priori information to initialize the anchor frame size. Thus, we could break through the limitation of the scale and receptive field of fixed frames, minimize the limitation and interference of the set a priori information to the classification and regression network, avoid the introduction of multi-layer hyperparameter information which is difficult to optimally adjust, and ensure the computational efficiency of the classification and regression network. 

#### 3.1.5. The Loss Function

For all the heads introduced above, they receive an input of tensor in the shape of 25 × 25 × 256 after the process of similarity matching. For the final output, the classification head generates a classification feature map $F_{cls}\in R^{H\times W\times 2}$ and the regression head generates a regression feature map $F_{reg}\in R^{H\times W\times 4}$, where *W* represents the width of the output feature map and *H* represents the height. In our case, they would be the same as the shape of the input tensor which is 25.

For the regression head, it outputs a feature map of size 25 × 25 with 4 channels, which records the distance from each corresponding location point to the 4 sides of the bounding box. This is noted as t(i,j) which is a *4D* vector (l,t,r,b), and could be calculated as follows:

(11) $$\begin{array}{l} t_{0}(i,j)=l=x-x_{0},t_{1}(i,j)=t=y-y_{0}\\ t_{2}(i,j)=r=x_{1}-x,t_{3}(i,j)=b=y_{1}-y \end{array}$$

where (x,y) represents the coordinates of the location of the search region corresponding to the point (i,j), and $\left(x_{0},y_{0}\right)$ and $\left(x_{1},y_{1}\right)$ represent the coordinates of the left-top and right-bottom corners of ground truth. The regression loss could be calculated by the following formula:

(12) $$L_{reg}=\frac{1}{\sum_{\left(i,j\right)}I_{\left(i,j\right)}}\sum_{\left(i,j\right)}I_{\left(i,j\right)}\times IOU[T_{\left(i,j\right)},t_{\left(i,j\right)}]$$

where $T_{\left(i,j\right)}$ represents the ground truth bounding box. *IOU* represents the common cross-merge ratio loss function, which is used to calculate the *IOU* loss between $T_{\left(i,j\right)}$ and $t_{\left(i,j\right)}$, and I(i,j) is a judgment function, the value of it would be 1 when the sample point lies within the ground truth box, otherwise, it is 0.

For the classification head, it outputs a feature map of size 25 × 25 with 2 channels, which records the information of foreground and background at each point (i,j) in a *2D* vector. The classification loss could be calculated using the method of cross-entropy loss:

(13) $$L_{cls}=0.5\times BCELoss(\delta_{pos},I)+0.5\times BCELoss(\delta_{neg},I)$$

where $\delta_{pos}$ and $\delta_{neg}$ represent the foreground and background scores stored in the *2D* vector for each corresponding position in the search region. I represents the ground truth of the classification label at the location, and BCELoss represents the binary cross-entropy loss function. 

For the centrality head, it outputs a feature map of size 25 × 25 with 1 channel called the centrality feature map $F_{cen}\in R^{H\times W\times 1}$, which records the centrality score C(i,j) of the corresponding position. The centrality score could be calculated by the formula:

(14) $$C(i,j)=I\times \sqrt{\frac{\min(l,t)}{\max(l,t)}\times \frac{\min(r,b)}{\max(r,b)}}$$

where I is a judgment function, the value of it would be 1 when the sample point is in the foreground and lies within the ground truth box, otherwise, it is 0. The centrality loss is

(15) $$L_{cen}=\frac{1}{\sum_{\left(i,j\right)}I_{}}\sum_{I=1}C(i,j)\times \log R(i,j)+(1-C(i,j))\times \log(1-R(i,j))$$

where C(i,j) represents the predicted centrality score for a specific location and R(i,j) represents the actual centrality score for this position.

In summary, the overall loss function of the algorithm is as follows:

(16) $$\;L=L_{cls}+\alpha_{1}L_{cen}+\alpha_{2}L_{reg}$$

where $L_{cls}$, $L_{cen}$, and $L_{reg}$ represent the classification loss, centrality loss, and regression loss, respectively. $\alpha_{1}$ and $\alpha_{2}$ are used as the weight hyperparameters to adjust the network and are set to 1 and 3, respectively, during the training process based on experimental and experiential evidence. 

## 4. Experiments and Discussion

### 4.1. Experiments Settings

#### 4.1.1. Experiments Environment and Parameter Settings 

The experimental environment of the algorithms in the paper is set up as follows: the CUDA version is 11.8, and the Python 3.7 + pytorch 1.13 programming framework is used to train and verify the algorithm performance. The hardware platform configuration used is AMD Ryzen5 5600 @4.40 GHz processor for CPU and Nvidia GeForce RTX3080 for GPU, the total memory capacity is 16GB.

The parameters of the training process are initialized as follows: the tracker is trained by applying the SGD optimizer with random batch gradient descent, the batch size is set to 12, and the training result of the ResNet-50 network on ImageNet [49], a large computer vision image dataset, is used as the pre-trained weight model for the backbone network. The following strategy is used for learning rate setting: in the process, the learning rate modification strategy is set to weight decay, and the first 5 iterative processes are trained using a warm-up [50] strategy, where we initialize the learning rate at 0.001 and subsequently increase by 0.001 for each iteration. The model is trained with the strategy of a gradient descent of the learning rate after the warm-up. A total of 20 rounds of iterative processes are performed and the overall training time of the network is 65 h.

#### 4.1.2. Datasets and Pretreatment

We train our SiamHAS network using the COCO [51], GOT-10K [52], and VID datasets as training sets with a sample size of 400,000 frames per round. To evaluate the performance in generalization and robustness of our algorithm from multiple perspectives, we use UAV123 [53] and OTB100 [54] datasets as test sets. A total of 100 video sequences containing various challenging scenes are available in the OTB100 dataset, and the UAV123 has 123 video sequences of aerial scenes, including multiple challenge categories such as scale transformation, occlusion, and viewpoint transformation in aerial scenes; it is the most frequently used test dataset for target tracking in aerial scenes.

**Figure 7:**
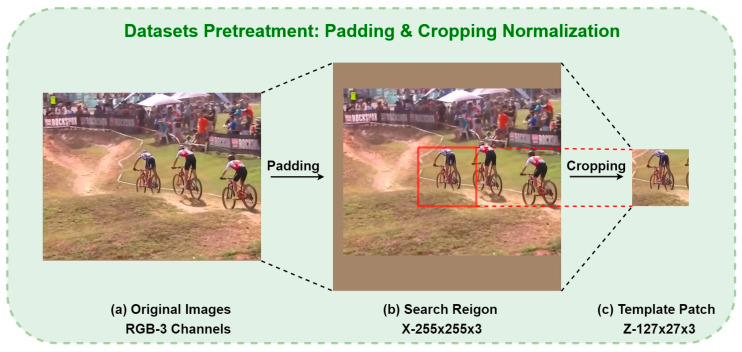
The process of datasets pretreatment: padding and cropping normalization. The (**a**) original images are adjusted through the padding process to be 255 × 255 as the inputs of the (**b**) search region branch X, and then are cropped to 127 × 127 as the inputs of the (**c**) template patch Z branch using the ground truth information.

We use the method of padding and cropping normalization to achieve scale-normalized preprocessing for images of different sizes in the datasets. The original images with a resolution less than 255 × 255 are filled with edge pixels to be the desired input resolution and used as the search region branch input of the network. Meanwhile, the ground truth information that is read from the Jason file is used as a basis to cut out the 127 × 127 area that contains most of the target’s information in the original picture, and then it is used as the template branch input. The purpose is to normalize the input and improve the training efficiency. Since the network structure will transform the input image into a smaller resolution 256 × 256 feature map, the normalized dataset will undoubtedly retain more target feature information and enhance the training speed of the algorithm and the effectiveness of the model. The following Figure 7 shows the detailed process of pre-processing described in the previous section.

### 4.2. Evaluation Metrics

**Figure 8:**
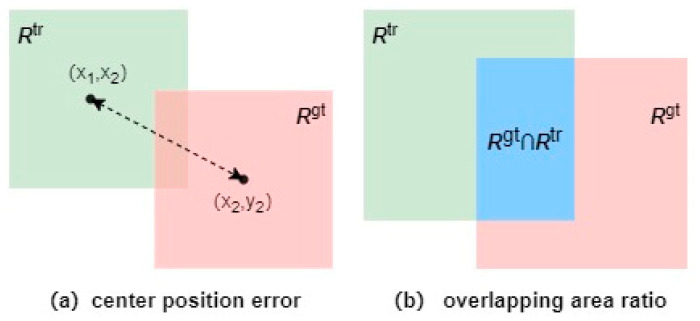
Visual presentation of (**a**) center position error and (**b**) overlapping area ratio. The centroid error between the target template and the search area prediction output and the overlapping area ratio of the two areas are used as the core evaluation calculation indicators.

The frequently used evaluation method for target tracking which is called one-pass evaluation (OPE) is generally used to initialize the first frame with the information in the ground truth. Then, the tracker could obtain the average accuracy and precision in the following tracking process. The common evaluation parameters for target tracking mainly include center position error and overlapping area ratio. The details will be described in the following subsections. Here Figure 8 shows a visual interpretation of the two kinds of evaluation metrics introduced above.

#### 4.2.1. Precision 

Precision is a common evaluation metric for single target tracking tasks. Precision is defined as the percentage of the frames where the Euclidean distance between the centroid of the target location generated by the tracker and the centroid of the manually labeled target is less than a set threshold. Usually, the threshold is set to 20 pixels, and the Euclidean distance within 20 pixels is regarded as the criterion for successful tracking. Specifically, the Euclidean distance calculation method between them is

(17) $$\rho =\sqrt{{\left(x_{2}-x_{1}\right)}^{2}+{\left(y_{2}-y_{1}\right)}^{2}}$$

Different thresholds are chosen as the criteria for judging the accuracy requirements, so different percentages of the frames that meet the thresholds can be obtained, and a precision curve can be drawn. 

#### 4.2.2. Success Rate

**Figure 9:**
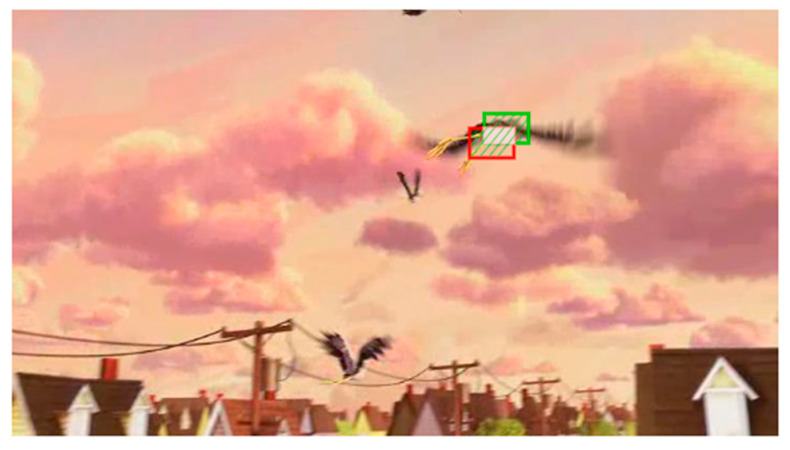
Visual presentation of the process of computing overlapping area ratio.

The success rate is determined by calculating the intersection ratio of the pixels in the region of the prediction box and the ground truth box, as shown in Figure 9 below. This is the ratio of the intersection area of the red box and the green box in the figure below to the total area surrounded by the two boxes.

Specifically, the overlap score OS is calculated as:

(18) $$OS=\frac{\left|x\cap y\right|}{\left|x\cup y\right|}$$

The bounding box generated by the tracker is recorded as x, the ground truth box is recorded as y, and |·| indicates the number of pixels in the region. When the OS of a frame is above the set threshold, it could be regarded as successful tracking, and the success rate is calculated by the percentage of the total successful frames to all the frames. The value range of OS is 0~1, so a success rate evaluation curve could be drawn.

### 4.3. Performance Comparison

#### 4.3.1. UAV123 Benchmark

The UAV123 benchmark is composed of 123 image sequences filmed by an aerial vehicle. It consists of various challenging characteristics, including tiny targets, low resolution, occlusion, and so on. Success and precision plots are the two kinds of evaluation methods for the trackers. For precision plots, the threshold is set to less than 20 pixels to judge if the specific frame meets the requirement. Precision refers to the percentage of frames whose CLE meets the requirements in the total frames. The formulas are as follows.

(19) $$\begin{array}{l} CLE=\sqrt{{\left(x_{pr}-x_{gt}\right)}^{2}+{\left(y_{pr}-y_{gt}\right)}^{2}}\\ f=\{\begin{array}{c} 1,CLE<20\\ 0,CLE\geq 20 \end{array}\\ Precision=\frac{\sum_{i=1}^{N}f}{N} \end{array}$$

In the formulas, (x_pr_, y_pr_) and (x_gt_, y_gt_) refer to the center position of the prediction box and ground truth, respectively. For success rate, the overlap ratio S threshold is set to larger than 0.5 to judge if the specific frame meets the requirement and can be regarded as successful tracking. The success rate is calculated by the percentage of the number of frames whose overlap ratio S meets the requirements in the total frames. The overlap represents the IOU of the prediction box and the ground truth of each frame. The final result takes the average value of all the frames. The formulas are as follows.

(20) $$\begin{array}{l} S=IOU\frac{\left|R_{pr}\cap R_{gt}\right|}{\left|R_{pr}\cup R_{gt}\right|}\\ f=\{\begin{array}{c} 1,IOU\geq 0.5\\ 0,IOU<0.5 \end{array}\\ Precision=\frac{\sum_{i=1}^{N}f}{N} \end{array}$$

The success plots and precision plots of the one-pass evaluation on the trackers are shown in Table 1. Our proposed tracker SiamHAS is observed to obtain a success rate of 0.627 and a precision of 0.820. Compared with other Siamese series trackers such as SiamBAN, which also has a similar overall structure design, our SiamHAS obtains a large improvement of 2.3% and 2.5% in success rate and precision.

#### 4.3.2. Attribute-Based Discussion on UAV123

**Figure 10:**
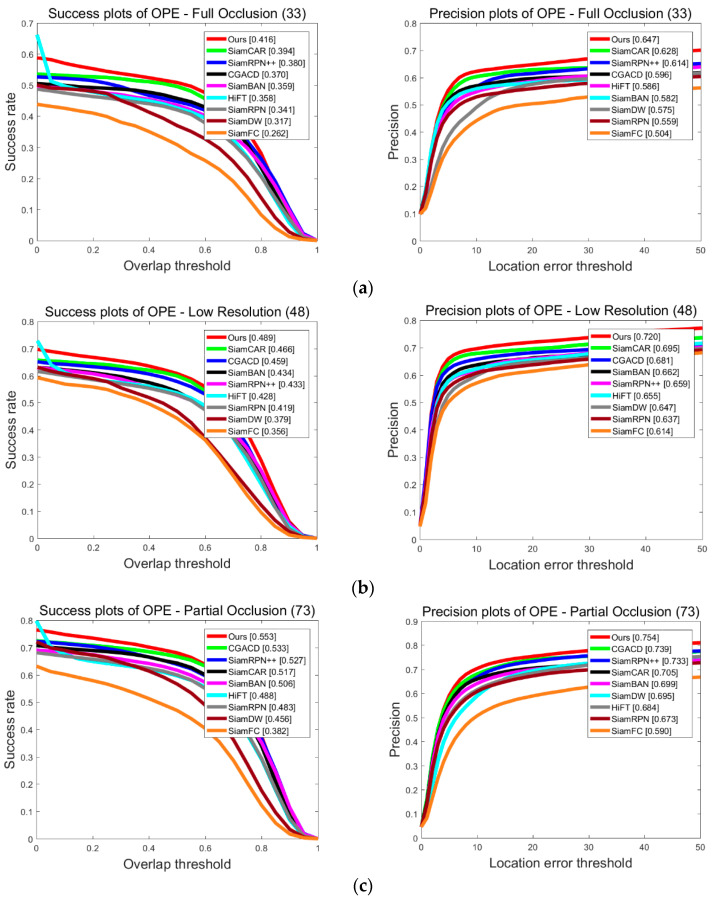

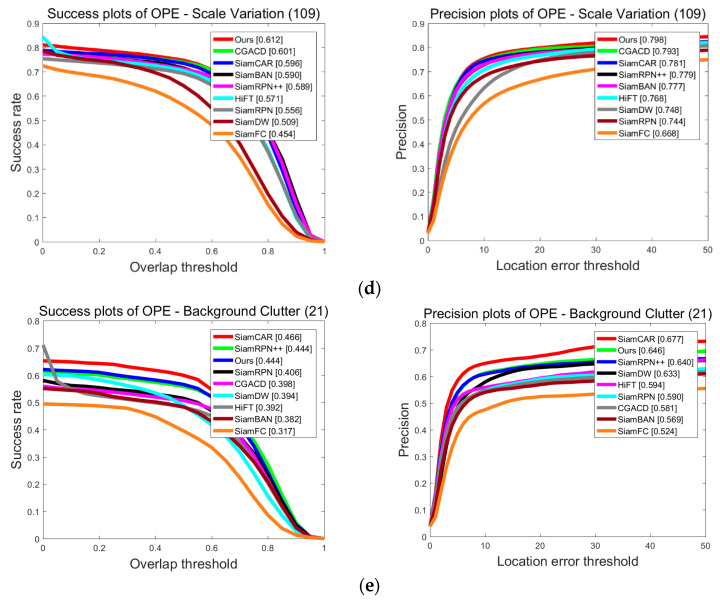
Attribute-based experiment results corresponding to 5 scenes on UAV123, including (**a**) full occlusion; (**b**) low resolution; (**c**) partial occlusion; (**d**) scaled variation; (**e**) background clutter.

To illustrate the performance improvement of the proposed method under challenging scenarios such as low resolution, occlusion, and scale variation, we conduct an attribute-based experiment on UAV123. The results of the attribute-based evaluation on UAV123 are shown in Figure 10, demonstrating that the proposed SiamHAS achieves the best evaluation results in all the attribute-based challenges. For success rate, the proposed SiamHAS obtains the best results, including full occlusion (0.416), low resolution (0.489), partial occlusion (0.553), and scale variation (0.612). In the standardized process of the one-pass evaluation for target tracking algorithms, for the success plots, the threshold of overlap ratio is set to 50 percent to judge whether a particular frame is tracked successfully or not, and the percentage of the frames judged to be successfully tracked to the total number of frames is defined as the success rate, which is recorded in the box on the right side corresponding to the different trackers, and the color ranking is constant and represents the success rate ranking of different trackers. Similarly, for the precision plots, the threshold of the location error is set to 20 pixels to judge whether a particular frame meets the requirements of expected precision or not. For the precision plot, the proposed SiamHAS also obtains the best performance among all the comparison attribute challenges mentioned above. Compared with the other Siamese series trackers such as SiamCAR, the proposed SiamHAS obtains significant performance improvement in full occlusion and low resolution challenges with 1.9% and 2.5% in precision, and 2.2% and 2.3% in success rate. Furthermore, it is worth noting that further attribute-based experiments show that the proposed method seems to show a degradation in the performance of background clutter scenarios. This might be caused by the fact that the proposed hierarchical attention mechanism does not balance well the effect of background noise while improving the saliency of the target. To sum up, the attribute-based discussion demonstrates that the proposed SiamHAS displays significant improvement in the performance of challenging aerial scenes and how to overcome the effect of background clutter will be a key consideration in our future work.

#### 4.3.3. Case Visual Comparison on UAV123

**Figure 11:**
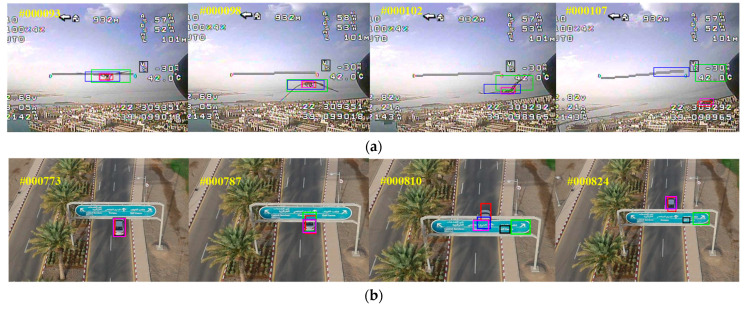

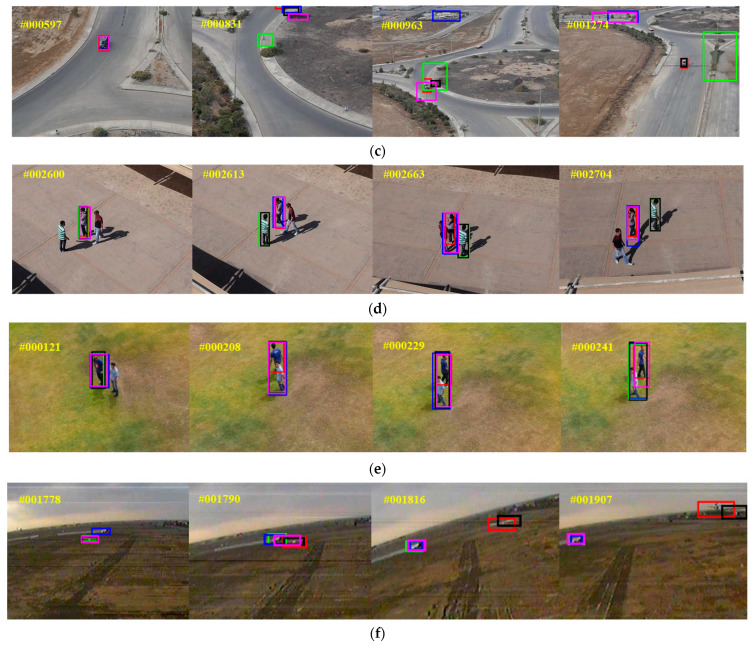
Case study of the proposed SiamHAS and several state-of-the-art trackers on the UAV123 dataset. From the top to bottom, the pictures show the visual case experiment results on the image sequences of (**a**) Bird1, (**b**) Car9, (**c**) Car14, (**d**) Group1, (**e**) Person11, and (**f**) Uav1. Red—Ours, Green—SiamCAR, Blue—SiamRPN++, Black—SiamRPN, Pink—SiamFC.

To intuitively show the improvement of our proposed tracker SiamHAS in coping with challenging scenes such as low resolution, occlusion, and scale variation, we choose six cases from the UAV123 dataset, that consist of the above issues. Results are then compared to other Siamese series trackers and several state-of-the-art trackers, and the case visual comparison is shown in Figure 11.

#### 4.3.4. UAV20L Benchmark

UAV20L is a dataset for aerial image and video analysis, which contains 20 long-time sequences of UAV images in different urban scenes. These image sequences cover a wide range of scenes, including city streets, parks, buildings, and natural landscapes, and are widely used as a benchmark to test the tracker’s long-time tracking performance. To further verify the robustness of the proposed SiamHAS in aerial scenarios, especially the performance when coping with long-time target tracking, we conducted experiments on the UAV20L benchmark to more comprehensively illustrate the performance improvement of the designed tracker in coping with aerial scenarios, and to intuitively represent the performance improvement of the tracker in optimizing the design for aerial scenarios. The success rate and precision of the one-pass evaluation on the trackers are shown in Table 2. Our proposed tracker SiamHAS is observed to obtain a success rate of 0.573 and a precision of 0.745. Compared with the other state-of-the-art Siamese series trackers such as SiamAPN++ [55], our SiamHAS obtains an improvement of 1.3% and 0.9% in success rate and precision. From the experimental results, we could draw the conclusion that the proposed SiamHAS tracker outperforms other state-of-the-art trackers in aerial long-term target tracking scenarios. Additionally, we have comprehensively evaluated the performance of the proposed tracker in aerial scenarios after the UAV20L benchmark experiment, which strongly supports the effectiveness of the innovative design and the improvement methods of the proposed tracker in adapting to the characteristics of aerial scenarios.

### 4.4. Quantitative Analysis

**Figure 12:**
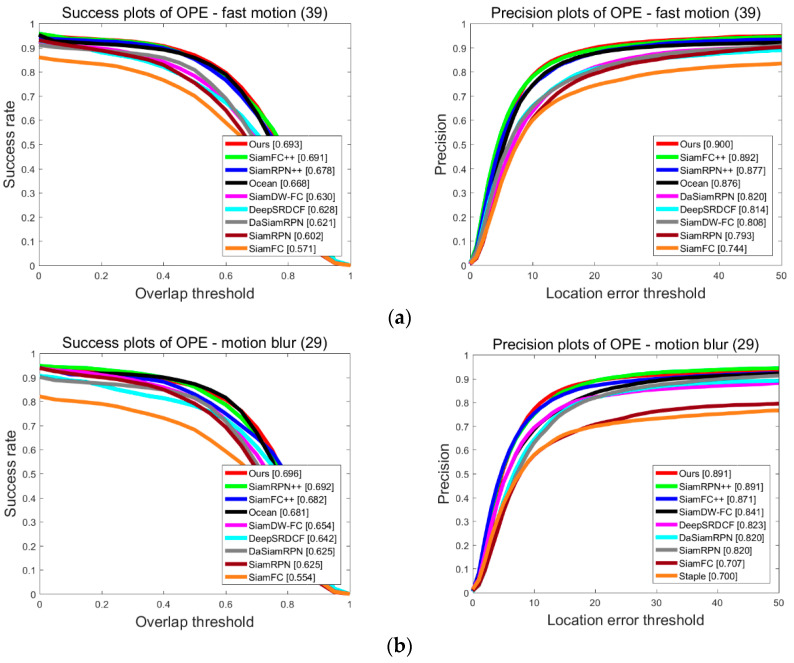

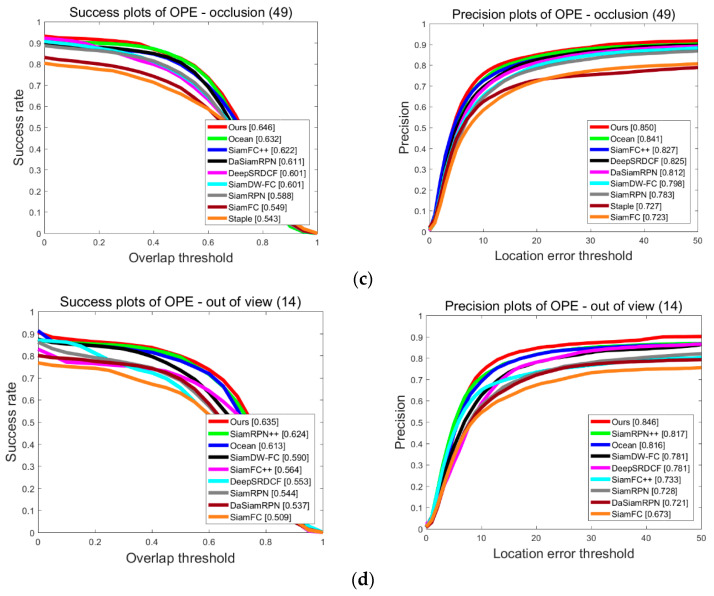
Experimental results corresponding to 4 typical scenarios on OTB100, including (**a**) fast motion; (**b**) motion blur; (**c**) occlusion; (**d**) out of view.

To validate the generalization ability of our SiamHAS in non-aerial scenarios for other types of challenges, we choose four typical scenarios in the OTB100 dataset including (a) fast motion, (b) motion blur, (c) occlusion, and (d) out of view for the attribute-based comparison experiments with some other state-of-the-art algorithms. Figure 12 displays the attribute-based evaluation and demonstrates that the proposed SiamHAS achieves the best performance compared with the other trackers that participate in the comparison on the attributes-based challenges. SiamHAS greatly improves the performance, especially in the two key scenes of occlusion and out of view. 

### 4.5. Discussion and Ablation Studies 

#### 4.5.1. Case Discussion on OTB100

**Figure 13:**
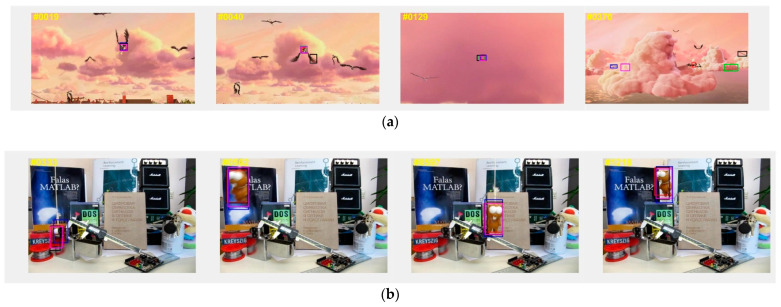

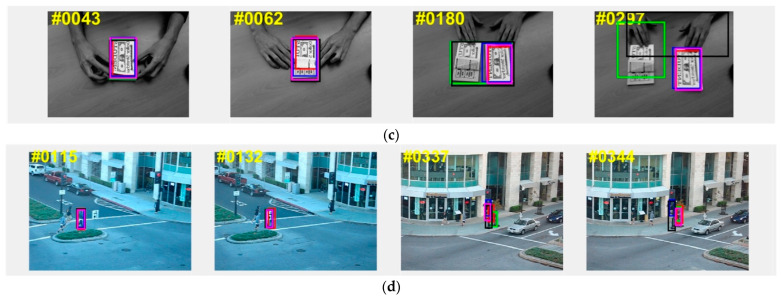
Case study on OTB100 for our SiamHAS and several state-of-the-art trackers. From the top to bottom, the pictures show the visual case experiment results on the image sequences of (**a**) Bird1, (**b**) lemming, (**c**) Coupon, and (**d**) human4. Red—Ours, Green—SiamCAR, Blue—SiamRPN++, Black—SiamRPN, and Pink—SiamFC.

To further validate the generalization capability of our tracker SiamHAS and intuitively demonstrate the performance improvement in coping with challenging scenarios such as occlusion, motion blur, and out of view, we choose four cases from the OTB100 dataset. The results are compared to several state-of-the-art trackers, such as SiamCAR, SiamRPN++, SiamRPN, and SiamFC, the visual comparison pictures are shown in Figure 13.

#### 4.5.2. Heat Map Experiment

**Figure 14:**
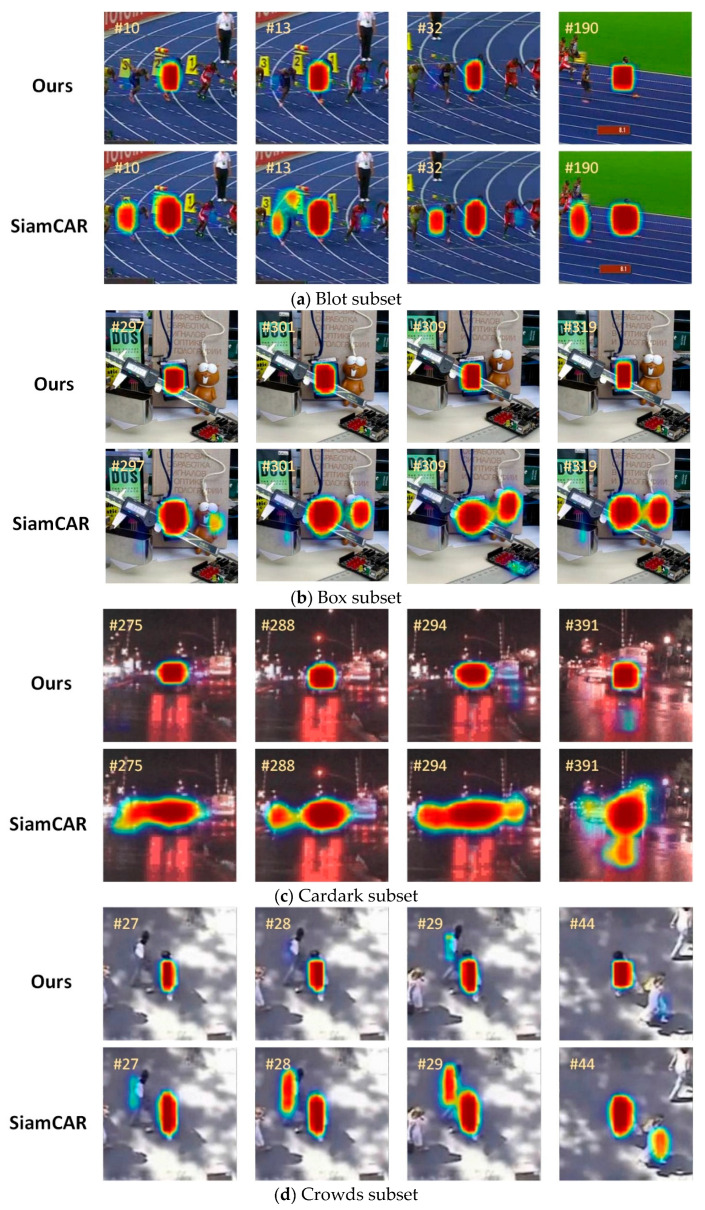

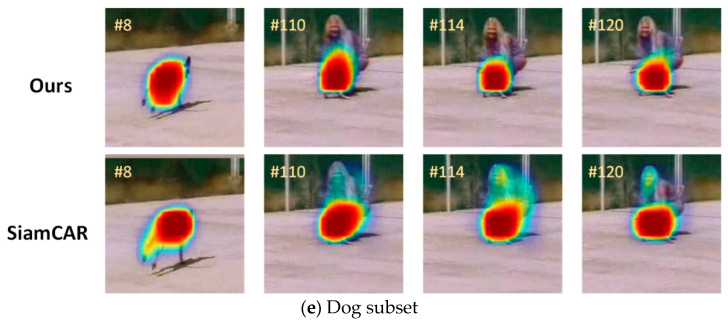
The heat map experiment visual results correspond to 5 scenes, including (**a**) blot subset; (**b**) box subset; (**c**) cardark subset; (**d**) crowds subset; (**e**) dog subset.

To more intuitively show the comparison between the other state-of-the-art Siamese series trackers’ and our SiamHAS’ focusing region and show the performance improvement of our tracker in coping with complex scenes such as similar object interference and motion blur, we perform a comparison-based heat map analysis with the recent and related approach SiamCAR under equal conditions. Specifically, we choose five cases in the OTB100 dataset, and the areas that draw the attention of the trackers are shown in Figure 14.

#### 4.5.3. Modules Ablation Experiment

To provide a deeper insight into how each of the proposed modules contributes to the overall network performance improvement, we carry out an ablation study on multiple benchmarks. The intention of this is to show how our proposed modules contribute to the performance of the tracker, and how much performance improvement every module could bring to the tracker. As shown in Table 3, we use the symbol √ to indicate the specific block has been added to the tracker architecture; × represents the opposite.

## 5. Conclusions

In our present article, we propose a novel Siamese tracker SiamHAS to improve the shortcomings of tracking drift and tracking failure in challenging aerial scenarios such as occlusion, aspect ratio change, low resolution, and scale variation. The proposed HAS module adopts a hierarchical attention strategy, which can take advantage of semantic and spatial features to break through the limitations of the receptive field of traditional convolutional neural networks and achieve multi-layer feature enhancement. In the channel attention part, we achieve global feature enhancement in the channel dimension, and through the channel context-aware process, we make the model adaptively distribute the attention scores of each channel based on global modeling. Therefore, there is an information exchange between different channels, which makes each channel contain more global contextual information and achieves a more effective feature optimization. In addition, self-attention enhancement of deep information is achieved by multiple parallel multi-headed attention networks, which enhances the model’s capability to distinguish foreground and background while balancing deep and shallow feature information. Additionally, we have performed many analyses and experiments on UAV123, UAV20L, and OTB100 datasets, experiments based on attribute challenges, case discussion, visualization, ablation experiments, etc. The experiments show that SiamHAS obtains significant capability improvement in all challenges compared with several state-of-the-art Siamese trackers; it also shows some generalization ability in the dataset of non-aerial scenes. Compared with the Siamese series tracker SiamCAR, it shows significant performance improvement for aerial scenarios, which also verifies that the proposed method can effectively cope with challenging scenarios such as occlusion, aspect ratio change, low resolution, etc., without losing much performance in real-time while ensuring the accuracy and robustness of the algorithm. In the case of using a deeper feature extraction network, the algorithm in this paper maintains a frame rate above 45 FPS, which meets the requirement of real-time tracking.

## Figures and Tables

**Table 1:** UAV123 benchmark comparison table, the table shows the success rate and precision results of 9 trackers including ours. The proposed SiamHAS performs favorably against state-of-the-art trackers. The best method is shown in red, and the second is shown in green.

Tracker	Ours	CGACD	SiamCAR	SiamRPN++	SiamBAN	HiFT	SiamDW	SiamRPN	SiamFC
Success	0.627	0.620	0.615	0.611	0.604	0.589	0.582	0.536	0.478
Precision	0.820	0.815	0.804	0.804	0.795	0.787	0.776	0.772	0.697

**Table 2:** UAV20L benchmark comparison table, the table shows the success rate and precision results of 8 trackers including ours. The proposed SiamHAS performs favorably against state-of-the-art trackers. The best method is shown in red, and the second is shown in green.

Tracker	Ours	SiamAPN++	HiFT	SiamAPN	SiamFC++	SiamRPN	DaSiamRPN	SiamFC
Success	0.573	0.560	0.553	0.539	0.533	0.528	0.547	0.423
Precision	0.745	0.736	0.736	0.721	0.695	0.696	0.676	0.629

**Table 3:** Comprehensive evaluation results with or without the proposed blocks on multiple datasets. Success-U and PRE-U represent the success rate and precision on the UAV123 benchmark; Success-O and PRE-O represent the success rate and precision on the OTB100 benchmark. FPS represents the selected results on the GOT-10K benchmark. The best method is shown in red, and the second is shown in green.

NO.	CCA	TME	Success-U	PRE-U	Success-O	PRE-O	FPS
1	×	×	0.599	0.773	0.676	0.879	51
2	√	×	0.601	0.789	0.681	0.881	49
3	×	√	0.615	0.804	0.686	0.887	47
4	√	√	0.627	0.820	0.693	0.896	45

## Data Availability

Not applicable.
